# Synthetic, Spectroscopic and Biocidal Aspects of Heterobimetallic Complexes Comprising Platinum(II) and a Group Four or Fourteen Element

**DOI:** 10.1155/MBD.2000.1

**Published:** 2000

**Authors:** Kripa Sharma, R. V. Singh

**Affiliations:** Department of Chemistry University of Rajasthan Jaipur 302004 India

## Abstract

Heterobimetallic complexes with varying amines have been synthesized by the reaction of [Pt(C_2_H_8_N_2_)_2_]Cl_2_ with group four or fourteen organometallic dichlorides, viz., R_2_MCl_2_ and Cp_2_M'Cl_2_ in a 1:2 molar ratio in MeOH (where M=Si or Sn, M'= Ti or Zr and R=Ph or Me). These complexes have been characterized by elemental analysis, molecular weight determinations, magnetic measurements, conductance, IR, ^1^H NMR and electronic spectra. The spectral data suggest a square planar geometry for all the complexes. Conductivity data suggest that they behave as electrolytes. These monometallic precursors along with their complexes have been screened *in vitro* against a number of pathogenic fungi and bacteria to assess their growth inhibiting potential.


### SYNTHETIC, SPECTROSCOPIC AND BIOCIDAL ASPECTS OF HETEROBIMETALLIC COMPLEXES COMPRISING PLATINUM(II) AND A GROUP FOUR OR FOURTEEN ELEMENT

Kripa Sharma and R.V.Singh*

Department of Chemistry, University of Rajasthan, Jaipur 302004, India

### ABSTRACT

Heterobimetallic complexes with varying amines have been synthesized by the reaction of $[Pt(C_2H_8N_2)_2]Cl_2$ with group four or fourteen organometallic dichlorides, viz., $R_2MCl_2$ and $Cp_2M'Cl_2$ in a 1:2 molar ratio in MeOH ( where M=Si or Sn, M'= Ti or Zr and R= Ph or Me). These complexes have been characterized by elemental analysis, molecular weight determinations, magnetic measurements, conductance, IR, $^1H$ NMR and electronic spectra. The spectral data suggest a square planar geometry for all the complexes. Conductivity data suggest that they behave as electrolytes. These monometallic precursors along with their complexes have been screened *in vitro* against a number of pathogenic fungi and bacteria to assess their growth inhibiting potential.

### INTRODUCTION

Macrocyclic ligands and their metal complexes are attracting the attention of many scientists mainly because of their tremendous importance, their structural peculiarities and biological importance. Several polydentate macrocyclic ligands and their metal complexes have been reported [1-2]. The use of metals as templates in such reactions has led to the synthesis of a large number of metal complexes of macrocyclic ligands[3] and thus show a specific coordination geometry on the metallation.[4] Macrocyclic ligands are chemically interesting as they show metal exchange (transmetallation) reactions, which are useful for the synthesis of new metal complexes. By a mismatch of requirement of the metal ion on the one hand and macrocyclic on the other, new reactions at the imino centres are obtained[5,6]. A current review[7] on synthetic chlorophyll and haemoglobin reveal the importance of polyazamacrocycles as oxygen carriers. Transition metals and their complexes have great interest and form multiple complexes[8-10]. Transition metal complexes of nitrogen donor ligands have been studied in detail, on account of their stereochemistry and wide practical utility[11-12]. A wider range of organosilicon compounds[13] are being studied due to their importance in resin and liquid polymers[14,15]. Organotin and organosilicon compounds exhibit a broad spectrum of biological activity which includes bactericidal[16,17], fungicidal[18], antitumor[19] and acaricidal effects.

There is growing awareness associated with electrochemical, magnetic and spectroscopic studies of multimetallic complexes[20] and their stable complexes that are of biological interest. The synthesis of bimetallic complexes is interesting because they exhibit magnetic exchange between the two metal ions or tendency to undergo multielectron redox reactions[21]. Related properties have been observed in natural systems (e.g. enzymes) which are known to incorporate interacting metal centres. The complexes are also expected to be useful as homogeneous catalysts in a number of reactions[22]. Therefore, it is desirable to study the heterobimetallic complexes of such metals and particularly of platinum as such compounds are limited. We report herein the synthesis, characterization and biological properties of some new heterobimetallic complexes of platinum and a group four or fourteen element.

### MATERIALS AND METHODS

The chemicals used were of AR grades. These chemical and solvents were dried and purified by standard methods.

### Preparation of $[Pt(C_2H_8N_2)_2]Cl_2$ and $[Pt(C_3H_{10}N_2)_2]Cl_2$

$[Pt(C_2H_8N_2)_2]Cl_2$ and $[Pt(C_3H_{10}N_2)_2]Cl_2$ were prepared by dissolving $PtCl_2$ in hot methanol with dropwise addtion of 1,2 diaminoethane or 1,3 diaminopropane. Platinum chloride and the diamine were added in a 1:2 stoichiometric ratio. The reactions were carried out by stirring the reactants for 6-7 hours. The product obtained was dried in vacuo and repeatedly washed with methanol. The reactions proceed as follows:

$$PtCl_2+2C_2H_8N_2 \longrightarrow [Pt(C_2H_8N_2)_2Cl_2$$

$$PtCl_2+2C_3H_{10}N_2 \longrightarrow [Pt(C_3H_{10}N_2)_2Cl_2$$

### Preparation of Heterobimetallic Complexes

A weighed amount of $[Pt(C_2H_8N_2)_2]Cl_2$ or $[Pt(C_3H_{10}N_2)_2]Cl_2$ was introduced into a dry 100 ml round bottom flask. The solution in methanol was treated with group fourteen metal dichlorides $Ph_2MCl_2$, $Me_2MCl_2$ and $Cp_2M'Cl_2$. In each case a coloured product appeared immediately, the reaction mixture was kept overnight and stirred for 6-7 hours. The product was obtained at room temperature. The compounds so formed were filtered and recrystallized from methanol and dried under reduced pressure. The yield was 60-70%.

**Analytical Methods and Physical Measurements**

Infrared spectra of precursors and complexes were recorded on a Nicolet-Megna FT-IR 550 spectrophotometer in a KBr pellets. Conductivity measurements were carried out on a systronic model 305 conductivity bridge with a cell constant of $1 cm^{-1}$. Conductivity was measured in a $10^{-3}$ M DMF solution. Magnetic susceptibilities of the complexes were recorded on a vibrating sample magnetometer Model 155 at the RSIC, IIT Madras. Electronic spectra of the isolated complexes were measured in DMSO on a Varian Cary/2390 spectrophotometer. $^1H$ NMR spectra were recorded on a JEOL FX-90Q spectrometer in DMSO-$d_6$ using TMS as internal standard. Molecular weights were determined by the Rast Camphor method. Platinum, titanium, zirconium, tin and silicon were estimated gravimetrically as their oxides. Nitrogen was estimated by Kjeldahl's method and chlorine, by Volhard's method. The analytical and physical data of the isolated precursors and their complexes are summarised in Table-I

**Table I: Physical Properties and Analytical Data of Complexes**

| Complex | Colour | M.P. (°C) | Analysis(%) | | | | Mol.Wt. |
|---|---|---|---|---|---|---|---|
| | | | M Found (Calcd) | Pt Found (Calcd) | N Found (Calcd) | Cl Found (Calcd) | Found (Calcd) |
| $Pt(C_2H_8N_2)_2]Cl_2$ | Brown | 240(d) | - | 50.21 (50.51) | 14.19 (14.50) | 18.13 (18.35) | 367.20 (386.20) |
| $Pt(C_2H_6N_2)_2Cl_2Sn_2(CH_3)_4$ | Light Brown | 240 | 34.88 (34.92) | 28.68 (28.70) | 8.21 (8.24) | 10.40 (10.43) | 654.89 (679.65) |
| $Pt(C_2H_6N_2)_2Cl_2Si_2(CH_3)_4$ | Green | 130(d) | 11.23 (11.26) | 38.10 (39.13) | 11.19 (11.23) | 14.18 (14.22) | 478.57 (498.44) |
| $Pt(C_2H_6N_2)_2Cl_2Si_2(C_6H_5)_4$ | Light Green | 120 | 7.48 (7.52) | 25.09 (26.12) | 7.48 (7.50) | 9.46 (9.49) | 729.83 (746.72) |
| $Pt(C_2H_6N_2)_2Cl_2Ti_2(C_5H_5)_4$ | Brown | 150 | 12.94 (12.97) | 26.39 (26.42) | 7.54 (7.58) | 9.56 (9.60) | 715.91 (738.30) |
| $Pt(C_2H_6N_2)_2Cl_2Zr_2(C_5H_5)_4$ | Orange | 190 | 22.08 (22.11) | 23.61 (23.64) | 6.75 (6.79) | 8.57 (8.59) | 804.97 (824.93) |
| $Pt(C_2H_6N_2)_2Cl_2Sn_2(C_6H_5)_4$ | Light Brown | 260 | 25.55 (25.58) | 20.98 (21.02) | 5.99 (6.03) | 7.61 (7.64) | 908.98 (927.91) |
| $Pt(C_3H_{10}N_2)_2Cl_2$ | Brown | 220(d) | - | 46.82 (47.09) | 13.29 (13.52) | 16.83 (17.11) | 395.26 (414.26) |
| $Pt(C_3H_8N_2)_2Cl_2Sn_2(CH_3)_4$ | Light Brown | 240(d) | 33.51 (33.54) | 27.51 (27.56) | 7.86 (7.91) | 9.82 (10.01) | 688.62 (707.70) |
| $Pt(C_3H_8N_2)_2Cl_2Si_2(CH_3)_4$ | Brown | > 300 | 10.62 (10.66) | 37.01 (37.05) | 10.61 (10.64) | 13.43 (13.46) | 508.00 (526.49) |
| $Pt(C_3H_8N_2)_2Cl_2Si_2(C_6H_5)_4$ | Light Green | 135 | 7.21 (7.24) | 25.14 (25.17) | 7.19 (7.23) | 9.12 (9.15) | 756.51 (774.76) |
| $Pt(C_3H_8N_2)_2Cl_2Ti_2(C_5H_5)_4$ | Orange | 160(d) | 12.23 (12.49) | 25.18 (25.45) | 7.12 (7.31) | 9.01 (9.05) | 749.39 (766.30) |
| $Pt(C_3H_8N_2)_2Cl_2Zr_2(C_5H_5)_4$ | Light Green | 225(d) | 21.11 (21.38) | 22.63 (22.87) | 6.28 (6.56) | 8.10 (8.31) | 828.97 (852.98) |
| $Pt(C_3H_8N_2)_2Cl_2Sn_2(C_6H_5)_4$ | Grey | 213 | 24.64 (24.83) | 20.19 (20.40) | 5.62 (5.86) | 7.19 (7.41) | 932.51 (955.96) |

**RESULTS AND DISCUSSION**

The elemental analysis and spectral data suggested the formation of the precursors $[Pt(C_2H_8N_2)_2]Cl_2$ and $[Pt(C_3H_{10}N_2)_2]Cl_2$ along with the bimetallic complexes $[Pt(C_2H_6N_2)_2M_2(R)_4]Cl_2$ and $[Pt(C_3H_8N_2)_2M_2(R)_4]Cl_2$. These bimolar reactions can be represented by the following reactions:

$$[Pt(C_2H_8N_2)_2]Cl_2 + 2R_2MCl_2 \longrightarrow [Pt(C_2H_6N_2)_2M_2(R)_4]Cl_2 + 4HCl$$

$$[Pt(C_2H_8N_2)_2]Cl_2 + 2Cp_2M'Cl_2 \longrightarrow [Pt(C_2H_6N_2)_2M'_2(R)_4]Cl_2 + 4HCl$$

(Where, M=Si or Sn ; M'=Zr or Ti and R=Ph or Me)

These reactions were carried out at room temperature. The resulting coloured solids are insoluble in water and common organic solvents such as ethanol, methanol, chloroform and carbon tetrachloride but are soluble in DMF and DMSO without change in colour. Molecular weight determinations showed them to be monomeric. Magnetic measurements showed that they are diamagnetic. The molar conductance data for these complexes in distilled DMF ($10^{-3}$M) are very high (215-290 $ohm^{-1}mol^{-1}cm^2$) showed that they are 1:2 electrolytes in DMF.

### SPECTRAL STUDIES

### I R Spectra

A comparative study of the IR spectra of the precursors and their complexes confirmed the formation of heterobimetallic complexes with the proposed coordination pattern. Both the precursors exhibit a broad and strong band of medium intensity in the region 3260-3120 $cm^{-1}$ that can be attributed to the $\upsilon_{NH}$ vibrations. The primary amine bands are observed at higher frequency than the corresponding secondary amine[23] bands of the precursor. The IR spectra of the ligands show higher frequency bands that disappear in the spectra of corresponding complexes, indicating the deprotonation of this group on complexation. This band shifted to lower frequency in the case of bimetallic chelates, confirming the formation of a group four or fourteen metal-nitrogen coordination bond[24]. The δ(N-H) in the precursor does not shift after chelation with the group four or fourteen element. Two sharp absorption bands observed at *ca* 1068 $cm^{-1}$ and 1385 $cm^{-1}$ in both the precursors and their bimatallic chelates are due to υ(C-C) and υ(C-N)[25] modes, respectively. Bands attributed to υ(M-N) are found in the low energy region. We have assigned the bands υ(Si ←N)[26] at 585 $cm^{-1}$ and υ(Sn←N) at 416 $cm^{-1}$ .

### $^1$H NMR Spectra

The $^1$H NMR spectra of the precursors as well as their corresponding metal complexes have been recorded in DMSO-$d_6$ using TMS as the internal standard. This spectra gives some important information to conclude to the formation of heterobimetallic complexes. The spectra of precursors show a band due to primary amino (δ4.09-4.11 ppm) suggesting the formation of proposed macrocyclic ring. The signals attributed to the methylene protons (δ2.06-2.43 ppm) were also assigned. Coordination pattern of heterobimetallic complexes further substantiated by the appearance of signals (δ8.22-8.63 ppm) due to the secondary amino protons (C-NH) and aromatic protons (δ7.29-8.01 ppm).

### Electronic Spectra

The electronic spectra of the metal complexes were recorded at room temperature in distilled DMSO. Three d-d spin allowed transitions were observed, corresponding to the transitions from the three lower lying 'd' levels to the empty $d_{x^2-y^2}$ orbitals. $^1A_{1g}$ is ground state and excited states corresponding to the above transitions are $^1A_2$, $^1B_{1g}$ and $^1E_g$ in order of increasing energy. Three d-d bands are observed in the range 515-532 nm, 445-470 nm and 342-376 nm in these present platinum complexes which may be assigned to $^1A_{1g}\rightarrow{}^1A_{2g}$, $^1A_{1g}\rightarrow{}^1B_{1g}$ and $^1A_{1g}\rightarrow{}^1E_g$ transitions, respectively. These values are in close agreement with those reported earlier for the square planar complexes[27].

On the basis of above evidences, structures *(1)* and *(2)* may be proposed for the heterobimetallic complexes.

### BIOLOGICAL STUDIES

Biochemical applications have greater demand nowadays. Activities of fungi and bacteria on several compounds give more important information about complexes. So it prompted us to screen all the heterobimetallic complexes and precursors to find out which part of the molecule is actually responsible for its physiological activity.

### Antifungal Activity

Agar medium for growing of the fungi is prepared. For this purpose glucose, starch, agar-agar and 1000ml of water were mixed at $25\pm2^0$C. Compounds after being dissolved in 50, 100 and 200 ppm concentrations in methanol were mixed into the medium. The linear growth of the fungus was obtained by measuring the diameter of colony in Petri plates after four days and the percentage inhibition was calculated with the relationship (C-T) 100 $C^{-1}$, Where, C and T are the Diameter of the fungus colony in control and test plates, respectively.

### Antibacterial Activity

Determination of antibacterial activity was carried out by the inhibition zone technique. All the compounds were dissolved in dry methanol in 500 and 1000 ppm. Paper discs of Whatman No.1 paper with a Diameter of 5 mm were soaked in these solutions. These discs were placed on the nutrient agar medium (Peptone, Beef extract, NaCl and agar-agar) previously seeded with organisms in Pertri dishes and stored in an incubator at $30\pm1^0$C. The inhibition zone thus formed around each disc was measured (in mm) after 24 hours. Data for fungicidal and bactericidal activities are given in Tables II and III.

**Table II: Fungicidal Screening Data of Precursors and their Heterobimetallic Complexes (Percent growth inhibition after 96 hours at 25±2$^0$C)**

| Compound | *Alternaria alternata* | | | *Fusarium oxysporum* | | | *Macropnomina phaseolina* | | |
|---|---|---|---|---|---|---|---|---|---|
| | 50 | 100 | 200 | 50 | 100 | 200 | 50 | 100 | 200 |
| $Pt(C_2H_8N_2)_2]Cl_2$ | 25 | 34 | 41 | 26 | 33 | 40 | 23 | 34 | 40 |
| $Pt(C_2H_6N_2)_2Cl_2Sn_2(CH_3)_4$ | 37 | 47 | 52 | 35 | 36 | 55 | 40 | 43 | 57 |
| $Pt(C_2H_6N_2)_2Cl_2Si_2(CH_3)_4$ | 35 | 45 | 50 | 32 | 34 | 53 | 37 | 40 | 55 |
| $Pt(C_2H_6N_2)_2Cl_2Si_2(C_6H_5)_4$ | 52 | 65 | 73 | 54 | 69 | 79 | 55 | 65 | 76 |
| $Pt(C_2H_6N_2)_2Cl_2Ti_2(C_5H_5)_4$ | 29 | 36 | 43 | 28 | 34 | 42 | 25 | 37 | 42 |
| $Pt(C_2H_6N_2)_2Cl_2Zr_2(C_5H_5)_4$ | 32 | 42 | 44 | 37 | 40 | 44 | 29 | 38 | 48 |
| $Pt(C_2H_6N_2)_2Cl_2Sn_2(C_6H_5)_4$ | 58 | 69 | 78 | 59 | 73 | 84 | 63 | 72 | 80 |
| $Pt(C_3H_{10}N_2)_2Cl_2$ | 28 | 39 | 44 | 29 | 37 | 43 | 25 | 36 | 43 |
| $Pt(C_3H_8N_2)_2Cl_2Sn_2(CH_3)_4$ | 39 | 52 | 56 | 37 | 38 | 56 | 42 | 46 | 59 |
| $Pt(C_3H_8N_2)_2Cl_2Si_2(CH_3)_4$ | 36 | 48 | 53 | 34 | 35 | 53 | 38 | 44 | 58 |
| $Pt(C_3H_8N_2)_2Cl_2Si_2(C_6H_5)_4$ | 55 | 68 | 74 | 57 | 71 | 80 | 56 | 68 | 78 |
| $Pt(C_3H_8N_2)_2Cl_2Ti_2(C_5H_5)_4$ | 30 | 39 | 46 | 32 | 38 | 47 | 29 | 43 | 47 |
| $Pt(C_3H_8N_2)_2Cl_2Zr_2(C_5H_5)_4$ | 33 | 45 | 47 | 40 | 43 | 45 | 32 | 40 | 52 |
| $Pt(C_3H_8N_2)_2Cl_2Sn_2(C_6H_5)_4$ | 61 | 72 | 81 | 63 | 76 | 85 | 67 | 76 | 82 |
| Standard (Bavistin) | 87 | 100 | 100 | 85 | 100 | 100 | 85 | 100 | 100 |

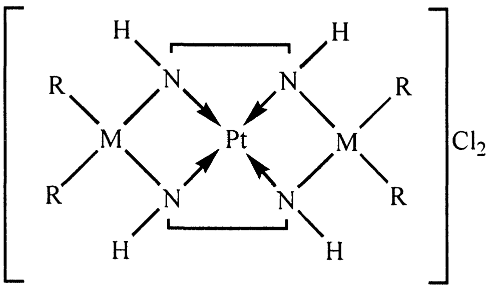

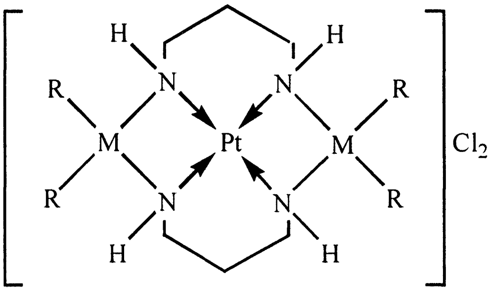

(1) (2)

**Table III: Bactericidal Screening Data of Precursors and their Heterobimetallic Complexes (Diameter of inhibition zone (mm) after 24 hours at 30±1$^0$C)**

| Compound | *Escherichia coli* | | *Staphylocoeus aureus* | | *Pseudomonas cepacicola* | |
|---|---|---|---|---|---|---|
| | 500 | 1000 | 500 | 1000 | 500 | 1000 |
| $Pt(C_2H_8N_2)_2]Cl_2$ | 3 | 6 | 4 | 7 | 2 | 3 |
| $Pt(C_2H_6N_2)_2Cl_2Sn_2(CH_3)_4$ | 6 | 8 | 6 | 9 | 4 | 6 |
| $Pt(C_2H_6N_2)_2Cl_2Si_2(CH_3)_4$ | 5 | 7 | 5 | 8 | 3 | 5 |
| $Pt(C_2H_6N_2)_2Cl_2Si_2(C_6H_5)_4$ | 7 | 9 | 7 | 10 | 6 | 8 |
| $Pt(C_2H_6N_2)_2Cl_2Ti_2(C_5H_5)_4$ | 4 | 6 | 5 | 8 | 3 | 4 |
| $Pt(C_2H_6N_2)_2Cl_2Zr_2(C_5H_5)_4$ | 4 | 7 | 5 | 8 | 4 | 4 |
| $Pt(C_2H_6N_2)_2Cl_2Sn_2(C_6H_5)_4$ | 9 | 11 | 10 | 11 | 8 | 12 |
| $Pt(C_3H_{10}N_2)_2Cl_2$ | 4 | 7 | 6 | 8 | 4 | 5 |
| $Pt(C_3H_8N_2)_2Cl_2Sn_2(CH_3)_4$ | 6 | 9 | 7 | 10 | 5 | 7 |
| $Pt(C_3H_8N_2)_2Cl_2Si_2(CH_3)_4$ | 6 | 8 | 6 | 8 | 4 | 6 |
| $Pt(C_3H_8N_2)_2Cl_2Si_2(C_6H_5)_4$ | 8 | 9 | 8 | 10 | 7 | 9 |
| $Pt(C_3H_8N_2)_2Cl_2Ti_2(C_5H_5)_4$ | 5 | 6 | 6 | 8 | 4 | 5 |
| $Pt(C_3H_8N_2)_2Cl_2Zr_2(C_5H_5)_4$ | 6 | 6 | 5 | 8 | 4 | 6 |
| $Pt(C_3H_8N_2)_2Cl_2Sn_2(C_6H_5)_4$ | 10 | 11 | 11 | 11 | 10 | 11 |
| Standard (Streptomycin) | 17 | 18 | 15 | 17 | 18 | 17 |

### RESULTS

The results show that there is a considerable increase in the toxicity of the complexes as compared to the precursors. It was important to note that complexes show more inhibitory effects and inhibit the growth of fungi and bacteria to a greater extent than the precursors and that the activity increases at higher concentrations. This can be well ascribe to Tweedy's chelation theory[28]. The results reveal that the toxicity of the bulkier group complexes is greater than that of the other precursors and complexes. The pathogens secreting various enzymes which breakdown the activities appear to be especially susceptible to inactivation by the ion of the complexes. The complexes facilitate their diffusion through the lipid layer of the spore membrane to the site of action and ultimately kill them by combining with -NH group of certain cell enzymes. According to Lawrence et al[29] the biocidal properties of complexes against various micro organisms depend on the impermeability of the cell. The bactericidal activity of complexes were more towards gram positive strain as compared to negative strain. Generally tin compounds are more active than silicon ones but in the case of phenylsilicon compounds silicon is more active than methylltin compounds due to the presence of bulkier groups. The precursors and their heterobimetallic complexes were tested for the *in vitro* growth inhibitory activity against pathogenic fungi *Alternaria alternata, Fusarium oxysporum, Macrophomina phaseolina* and bacteria *Escherichia coli*, *Staphylococcus aureus* and *Pseudomonas cepacicola*. The results recorded from the biological activity were also further compared with the standard fungicide *Bavistin* and conventional bactericide *Streptomycin*.

**ACKNOWLEDGEMENT**

The authors are thankful to CSIR, New Delhi, India for financial assistance through grant number 01(1490)/EMR-II.

**REFERENCES**

1. S.G. Kang, M.S. Kim, D. Whang and K. Kim, *J.Chem. Soc. Dalton Trans.*, 853 (1994).
2. M. Shakir, S.P.Varkey and T.A. Khan, *Indian J.Chem*, **34A,** 72 (1995).
3. S.Chandra and R.Singh, *Indian J.Chem*, **34A,** 1003 (1995).
4. E.S. Eggleston and S.C. Jackels, *Inorg. Chem.* , **19,** 1593 (1980).
5. S.M. Nelson, C.V. Knox, M.McCann and M.G.B. Drew, *J..Chem.Soc., Dalton T*rans., 1669 (1981).
6. M.G.B.Drew, J.Nelson and S.M. Nelson, *J. Chem. Soc., Dalton Trans*, 1678 (1981).
7. M. Momentean and, C.A. Reed, *Chem.,Rev.,* **94,** 585 (1994) .
8. I. Haiduc, *Coord. Chem. Rev*., **99**, 253 (1990) and references their in.
9. M.J. Cleare, *Coord. Chem. Rev.,* **12** 349 (1974)
10. B.Singh, R.N. Singh and R.C. Aggarwal, *Polyhedron*, **4**, 401 (1985)
11. K. Dey, D.Bandyopadhyay, K.K. Nandi S.N. Poddar, G. Mukhopadhyay and G.B. Kauffman, *Synth. React. Inorg. Met-Org. Chem*., **22,** 1111 (1992) .
12. M. A. Ali and S.E. Livingstone, *Coord. Chem. Rev*., **13**, 101 (1974).
13. C. Saxena and R.V. Singh, *Phosphorus*, *Sulfur and Silicon*, **97**, 17 (1994).
14. H.Nagy Kovacs, A.D. Delman and B.B.Simms, *Polymer Sci.*, **4**, 1081 (1966).
15. Bazant V, V. Chalovsky and J. Rathousky, *J. Organosilicon Compounds,* (Czech Acad. Sci, Prague and Academic Press, New York), **2**, Parts 1 and 2 (1965)
16. T.M. Aminabhavi, N.S. Biradar, S.B. Patil, V.I. Roddabasangoudar and W.E. Rudzinski, *Inorg. Chim. Acta,* **107**, 231 (1985).
17. O.R. Klinumer, *Pflanzeschutzberichte,* **37**, 57 (1968).
18. L. Moens, H. Maraite, B. Mahieu, A. El. Khloufia, R. Willem and M. Gielen, *Main Group Met. Chem*. **15**, 275 (1992).
19. M.Gielen, P. Lelieveld, D. Devos and R. Willem, Metal-based Antitumour Drugs, Gielen, M. (ed.), Freund, 1992, Vol 2, p.95. London.
20. S. Wang, Garzon, C. King, J. Wang, J.P. Fackler Jr., *Inorg. Chem*., **28**, 4623 (1989).
21. D.E. Fenton, V. Casellato, P.A. Vigato and M. Vidali, *Inorg.Chim. Acta*, **62**, 57 (1982).
22. S.M. Nelson, F.S. Esho, A. Lavery and M.G.B. Drew, *J. Am. Chem. Soc*., **105**, 5693 (1983).
23. K. Nakamato, Infrared and Raman Spectra of Inorganic and Coordination Compounds, Willey, New York, P.**201** (1978).
24. K.S. Siddiqui, Aqra FMAM and S.A.A. Zaidi, *Transition. Met. Chem*., **18**, 420 (1993).
25. R.M. Silverstein, G.C. Bassler, T.C. Morrill, *Spectrometric Identification of Organic Compounds*, **4,** 128 (1981).
26. E.A.V. Ebsworth and M.J. Mays, *J. Chem. Soc*., 3750 (1964).
27. H.B. Gray and C.J. Ballhousen, *J.Am. Chem. Soc*., **85**, 260 (1963).
28. B.G. Tweedy, *Phytopathology*, **55**, 910 (1964).
29. P.G. Lawrence, P.L. Harold and O.G. Francis, *Antibiotic and Chemotherapy*, **5**, 1597 (1980).

**Received: August 24, 1999 - Accepted: September 27, 1999 -**
**Received in revised camera-ready format: October 21, 1999**

